# Case Report: Endoscopic dual-port surgery via the previous resection cavity for recurrent glioblastoma

**DOI:** 10.3389/fsurg.2026.1794899

**Published:** 2026-03-10

**Authors:** Kento Takahara, Takuya Kitamura, Nobuhiro Yamada, Hirotsugu Nogawa, Masahiro Ogino

**Affiliations:** 1 Department of Neurosurgery, Ashikaga Red Cross Hospital, Tochigi, Japan; 2 Department of Neurosurgery, Keio University School of Medicine, Tokyo, Japan

**Keywords:** dual-port, endoscope, real-time neuronavigation, recurrent glioblastoma, resection cavity

## Abstract

**Introduction:**

Although endoscopic techniques have become increasingly common in neurosurgery, true multi-port surgeries for intracranial lesions remain rare. Unlike laparoscopic or thoracoscopic procedures, intracranial surgery often requires traversal of normal brain parenchyma, limiting the creation of multiple access routes. However, after resection of intraparenchymal tumors, a postoperative cavity frequently remains and may serve as a potential working space for endoscopic manipulation. The feasibility of using such cavities for multi-port endoscopic tumor resection has not yet been established.

**Case description:**

A 72-year-old man had previously undergone gross total resection of a contrast-enhanced lesion, followed by radiochemotherapy for a right frontal glioblastoma. Ten months later, a small, locally recurrent enhancing lesion developed along the posterior wall of the resection cavity. Given the patient's advanced age, comorbid diabetes mellitus, and the superficial location of recurrence, a minimally invasive multi-port endoscopic resection was planned. Limited reopening of the original skin incision was performed without removal of the bone flap. Two ports were created: one at the edge of the craniotomy and another through a 5-mm hole in the bone flap directly above the resection cavity. Tumor resection was performed under endoscopic visualization using an ultrasonic aspirator with real-time neuronavigation guidance. The multi-port configuration enabled stress-free bimanual manipulation without instrument interference. Near-total resection was achieved, with a residual enhancing tumor <1 cm in size. The postoperative course was uneventful.

**Conclusions:**

This case demonstrates the feasibility of a minimally invasive multi-port endoscopic approach utilizing a pre-existing resection cavity for recurrent intracranial lesions. When a superficial and accessible postoperative cavity is present, this strategy may reduce surgical invasiveness and wound-related complications while providing a favorable operative environment.

## Introduction

Minimally invasive endoscopic surgery is becoming increasingly common in neurosurgery. Endoscopy is generally well-suited for procedures involving pre-existing cavities, as demonstrated in laparoscopic surgery for the abdominal cavity and thoracoscopic surgery of the thoracic cavity. In neurosurgery, endoscopic procedures utilizing pre-existing cavities, such as the nasal cavity and ventricular system, are well established.

Multi-port surgery utilizing pre-existing cavities is commonly performed in laparoscopic and thoracoscopic procedures; however, its application to intracranial lesions remains quite limited (1–4). Conventional neuroendoscopic surgery is almost exclusively performed as a single-port procedure, which inevitably results in significant interference between the endoscope and the surgical instruments, leading to substantial technical limitations and the requirement for highly specialized skills.

In the field of neurosurgery, reports involving multiple ports have been increasing, mainly in the context of combined approaches for complex skull base lesions (5–8). However, these approaches add an alternative surgical corridor to address lesions that are difficult to manage through a single corridor and therefore differ fundamentally from the concept of multi-port surgery as practiced in laparoscopic or thoracoscopic procedures. The limited adoption of multi-port surgery in neurosurgery may be attributed to factors such as difficulty accessing intracranial cavities and the frequent need to traverse normal brain parenchyma, unlike in abdominal or thoracic cavities.

After the resection of intraparenchymal brain tumors, the postoperative cavity often remains at an anatomically accessible location, which is theoretically suitable for endoscopic manipulation. Nevertheless, there have been no reports describing endoscopic tumor resection utilizing this postoperative cavity, and it remains unclear whether such cavities are well suited for endoscopic surgery.

Although the role of surgical resection for recurrent glioblastoma (GBM) has not been definitively established, gross total resection of contrast-enhancing lesions has been reported to confer a survival benefit (9), and surgical intervention is therefore performed in selected cases. In most instances, the original skin incision is reused, and a repeat craniotomy with bone flap removal is performed. Repeated reopening of the same surgical site, often in previously irradiated tissues, is associated with an increased risk of wound-related complications (10). Moreover, when the recurrent lesion is small, repeat craniotomies may be considered excessively invasive. Despite these concerns, surgical options remain limited. To the best of our knowledge, there have been no reports describing minimally invasive resection for recurrent glioblastoma after prior craniotomy.

Here, we report a case of recurrent glioblastoma in which minimally invasive multi-port resection was successfully performed without removing the bone flap using an endoscope and the postoperative resection cavity created during the initial surgery.

## Case presentation

A 72-year-old man underwent standard treatment for a newly diagnosed right frontal glioblastoma 10 months earlier, consisting of maximal surgical resection followed by radiochemotherapy with fractionated irradiation (40 Gy with an additional focal boost of 15 Gy) and the alkylating agent temozolomide. During the initial surgery, the tumor was resected via a conventional craniotomy and gross total resection of the contrast-enhancing lesion was achieved.

**Figure 1:**
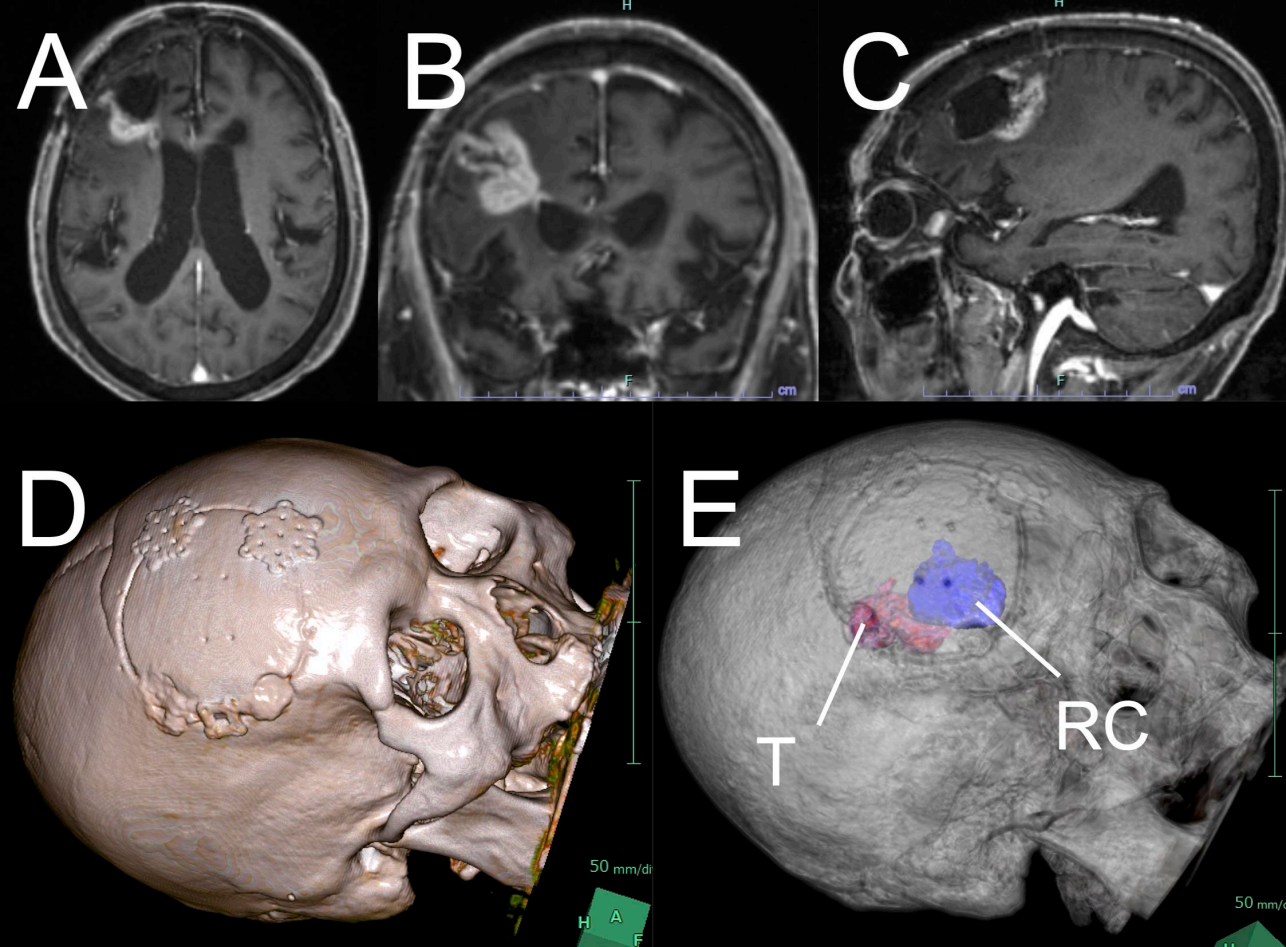
Preoperative image findings and simulation. Preoperative **(A)** axial, **(B)** coronal, and **(C)** sagittal contrast-enhanced MRI show a recurrent lesion in the posterior wall of the previous resection cavity. (**D, E**) Preoperative simulation revealed the lesion. Preoperative simulation showed the recurrent tumor along the posterior margin of the previous craniotomy and the previous resection cavity beneath the bone flap. T, tumor; RC, resection cavity.

During follow-up, magnetic resonance imaging (MRI) revealed a recurrent contrast-enhancing lesion localized to the posterior wall of the resection cavity. The lesion showed progressive enlargement over time (Figures 1A–C), accompanied by expansion of the surrounding high-intensity FLAIR signal. Although the patient exhibited no overt clinical deterioration, additional surgical resection was considered beneficial for tumor control, and a repeat surgery was planned.

Preoperative simulation demonstrated that the recurrent tumor was located almost entirely along the posterior margin of the previous craniotomy and that the resection cavity lay immediately beneath the bone flap (Figures 1D,E). Based on these findings, complete reopening of the previous surgical incision was not necessary.

Considering the patient's advanced age and history of diabetes mellitus, minimizing wound-related complications and surgical invasiveness was a priority. Therefore, a minimally invasive multi-port endoscopic procedure was planned, utilizing the postoperative resection cavity as a working space. The cavity was considered suitable for endoscopic visualization, and an endoscopic port was planned directly above the cavity.

### Surgical procedure

Surgery was completed under general anesthesia with the patient in a supine position and the head fixed in the neutral midline position. Neuronavigation and intraoperative monitoring of motor- and somatosensory-evoked potentials (MEP/SEP) were performed throughout the procedure.

**Figure 2:**
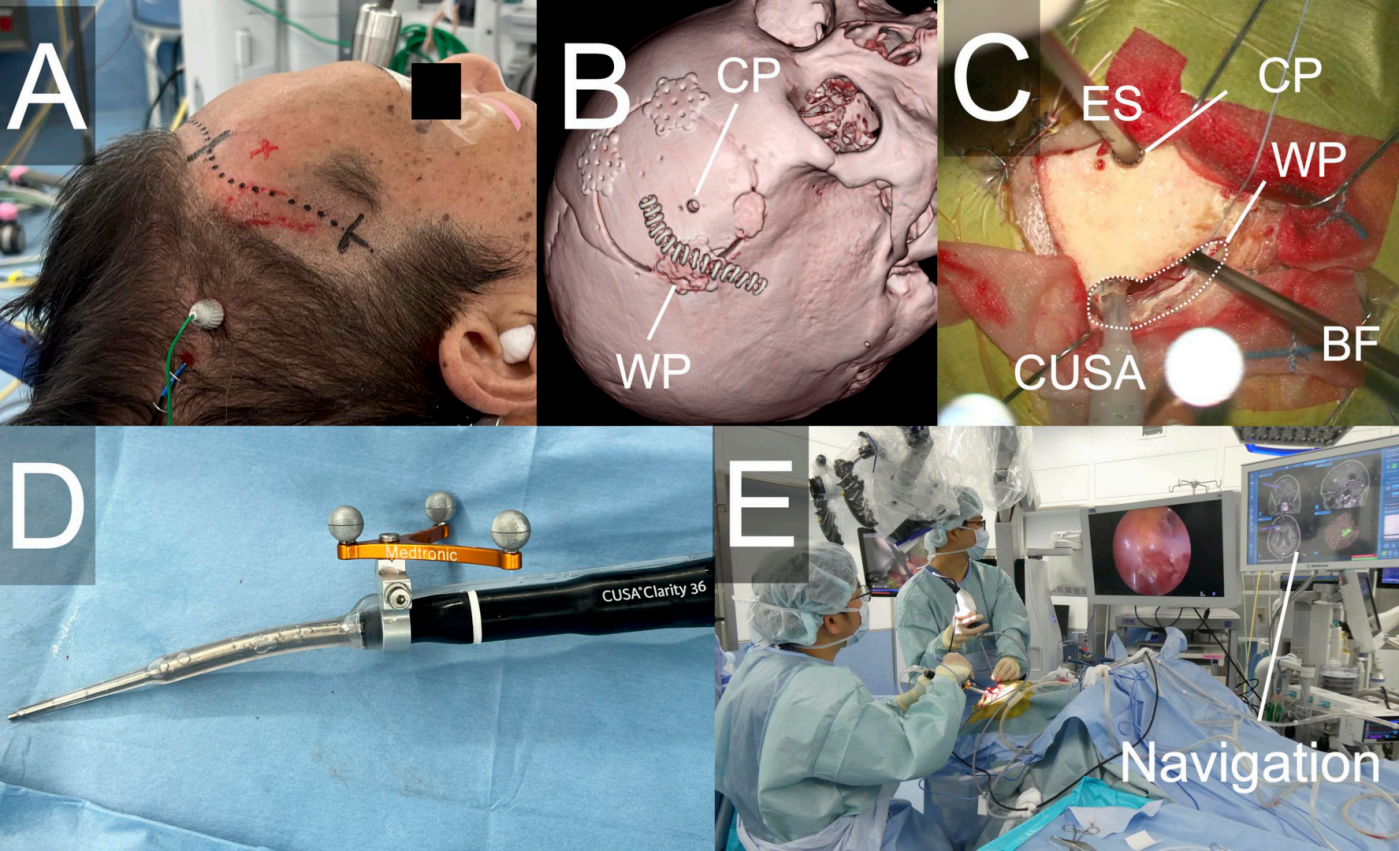
Operative setups and used instruments. **(A)** Skin incision utilizing part of the previous surgical incision. **(B)** Postoperative CT image showing the skin incision and the camera port. **(C)** Setup of the camera and surgical instruments during manipulation. **(D)** Optical navigation integrated with the CUSA. **(E)** Real-time navigation enabling simultaneous tumor resection and positional confirmation. CT, computed tomography; CP, camera port; WP, working port; ES, endoscope; BF, bipolar forceps.

A 6.5-cm portion of the original skin incision near the recurrent lesion was reopened to expose both the margin of the previous craniotomy adjacent to the recurrent tumor and the area directly above the resection cavity (Figures 2A,B). The titanium plate along the craniotomy edge was removed, and an additional bone measuring approximately 12 × 7 mm was drilled adjacent to the original burr hole, creating an enlarged bone opening of approximately 30 × 9 mm. This opening served as the working port. On the bone flap side, a 5-mm-diameter hole was drilled as the camera port (Figure 2C).

Under microscopic visualization, the adhesions of the brain tissue directly beneath the port at the craniotomy edge were carefully dissected to establish continuity with the resection cavity. Subsequently, an endoscope was introduced through the camera port. A 4-mm rigid endoscope (Karl Storz, Tuttlingen, Germany) was employed. Tumor resection was performed using a CUSA® Clarity ultrasonic surgical aspirator (Integra Life Sciences, Princeton, NJ, USA) under endoscopic visualization. Because anatomical landmarks within the resection cavity are limited, accurate localization of recurrent lesions was challenging. Therefore, optical navigation (StealthStation™ S7, Medtronic, Dublin, Ireland) integrated with the CUSA was utilized (Figure 2D), enabling real-time confirmation of the instrument tip position during tumor removal (Figure 2E).

**Figure 3:**
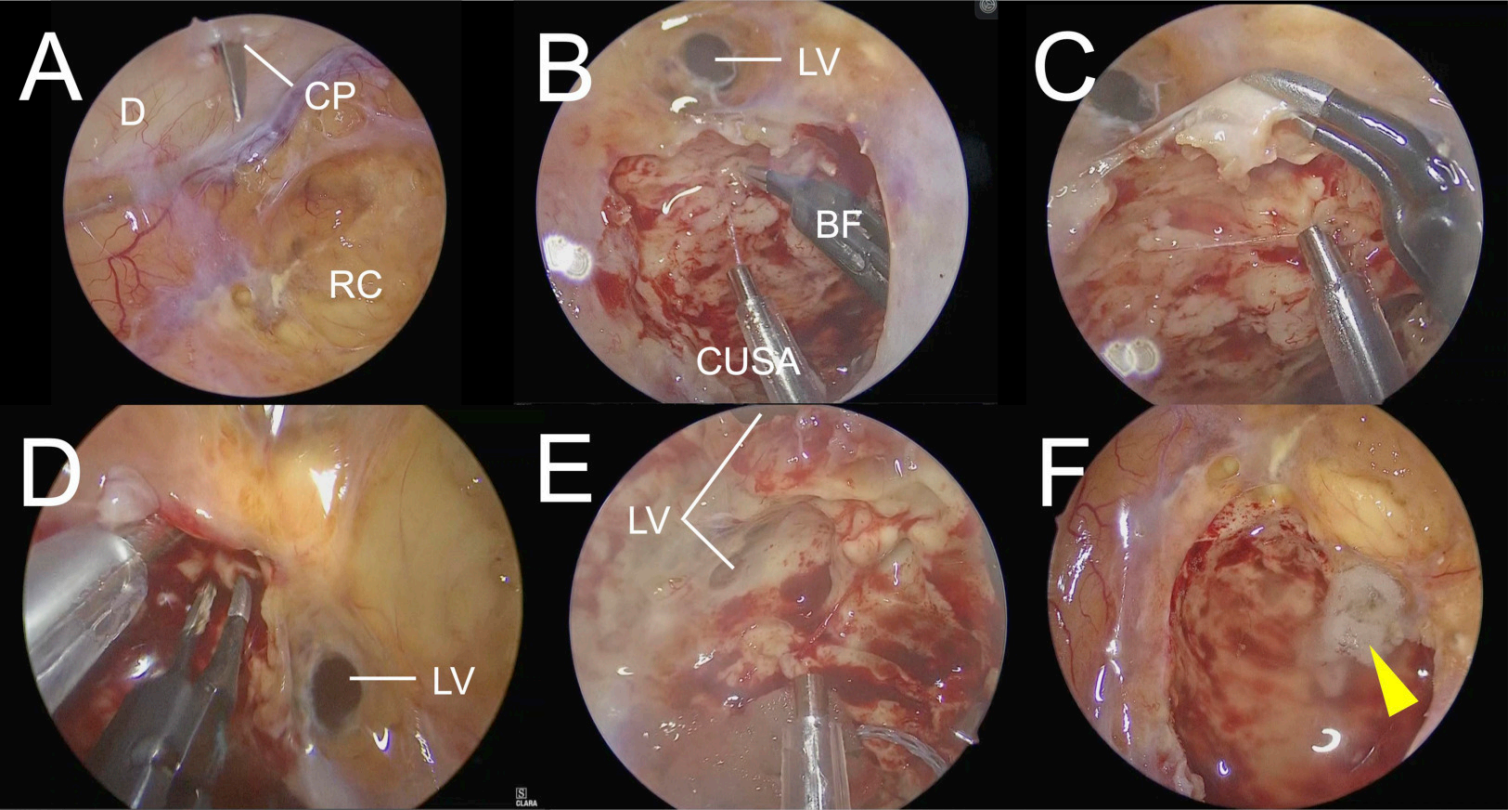
Endoscopic view during surgery. **(F)** The reconstructed ventricular wall is indicated by an arrowhead. D, dura mater; CP, camera port; RC, resection cavity; LV, lateral ventricle; BF, bipolar forceps.

When the endoscope was introduced through the working port, the camera port was positioned directly above the resection cavity (Figure 3A). The recurrent lesion was resected using a CUSA and bipolar forceps. The recurrent lesion was slightly greyish and relatively firm. Areas near the working port were visualized using a 30° endoscope (Figures 3B,C), and areas close to the camera port were pictured using a 0°endoscope (Figure 3D). Partial opening of the lateral ventricle occurred during tumor removal; therefore, the ventricular opening was intentionally kept minimal, leaving a thin layer of tumor tissue to avoid excessive ventricular exposure (Figure 3E). In this surgical setup, no interference between the endoscope and surgical instruments was encountered, allowing stress-free bimanual manipulation using bipolar forceps and CUSA.

The ventricular wall was reconstructed using oxidized regenerated cellulose (Surgicel®) and fibrin glue (Figure 3F). Based on endoscopic findings and navigation information, the enhanced lesion was completely removed. Dural reconstruction was performed using a dural substitute (DuraGen®; Integra Life Sciences, Princeton, NJ, USA), and the craniotomy edge was covered and fixed with a titanium plate before wound closure. The total operative time was 3 h and 33 min.

**Figure 4:**
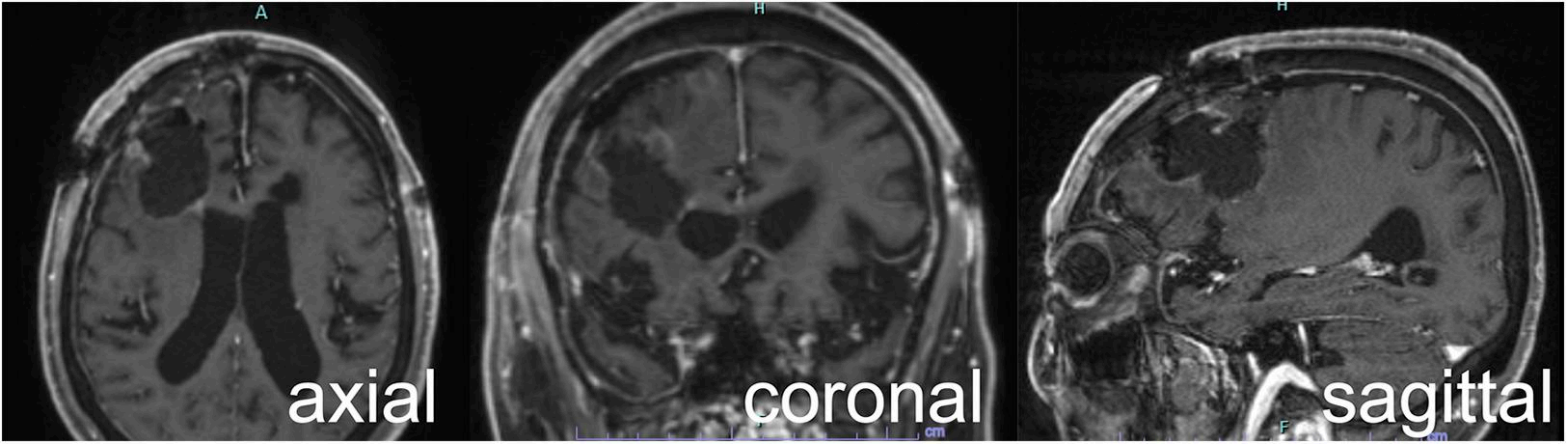
Postoperative contrast-enhanced MRI showing near-total resection of the enhancing lesion. MRI, magnetic resonance imaging.

The postoperative course was uneventful, and no new neurological deficits were observed. Postoperative MRI confirmed near-total resection with a residual enhanced tumor size of <1 cm^3^ (Figure 4). The patient was discharged on postoperative day 10. Histopathological examination confirmed a recurrent GBM.

## Discussion

To the best of our knowledge, this is the first report of a multi-port surgical approach using a pre-existing resection cavity for intracranial lesions. This approach demonstrated excellent compatibility with endoscopic visualization, providing a sufficient surgical field and enabling stress-free bimanual manipulations. In addition to reducing overall surgical invasiveness, this strategy may help decrease wound-related complications associated with repeated skin incisions and craniotomies, potentially representing a novel therapeutic option for selected cases.

Multi-port surgery may offer a new minimally invasive alternative in the field of neurosurgery, particularly when the resection cavity is relatively superficial. Most endoscopic procedures for intracranial lesions are performed using a single-port technique in which both the endoscope and surgical instruments are introduced through the same corridor. Unlike laparoscopic or thoracoscopic surgery, creating multiple independent access routes to intracranial lesions is difficult because accessing intraparenchymal tumors often requires traversing the normal brain tissue or other vital structures. Furthermore, the historical transition from conventional microscopic surgery may have contributed to the predominance of single-port strategies in neuroendoscopic surgeries. Reports of true multi-port approaches in neurosurgery are limited to specific situations, such as partial removal of gliomas or intraventricular lesions (1–3). In contrast to approaches for deep intraparenchymal or skull base tumors, this technique utilizes a relatively superficial and easily accessible cavity. The underlying concept is that the procedure can be simplified and made less invasive compared with conventional approaches.

However, when a pre-existing resection cavity is present near the cortical surface and is easily accessible, as in the present case, such a strategy becomes feasible. In single-port neuroendoscopic surgery, interference between the endoscope and surgical instruments remains a major technical limitation (11). In contrast, the multi-port technique used in this case markedly reduced this interference, resulting in a comfortable operative environment without procedural stress. The residual tumor was intentionally left in place to avoid excessive opening of the ventricle. Importantly, this approach allowed surgical manipulation comparable to that achieved with conventional techniques. The operative handling was similar to that experienced in laparoscopic and thoracoscopic surgeries. In addition, the panoramic view provided by the endoscope allowed wide visualization of the cavity.

Although the lateral ventricle served as an anatomical landmark in this case, a simple resection cavity alone often lacks reliable landmarks and may predispose surgeons to disorientation. Therefore, the integration of real-time neuronavigation was particularly valuable in compensating for this limitation and facilitating accurate lesion localization.

This approach may represent a useful option for surgery in patients with recurrent GBM and other recurrent intracranial lesions, particularly when excessive invasiveness and wound-related complications are avoided. The most suitable indications for this technique are cases in which a usable resection cavity exists at a superficial location adjacent to the target lesion. Conversely, this technique may not be appropriate when access is limited due to the absence of a superficial cavity, when the recurrent lesion is not adjacent to the cavity, or when high tumor vascularity is expected to make hemostasis challenging. While no studies have directly demonstrated that limiting the extent of reopening during reoperation reduces wound-related complications, minimally invasive surgery has been associated with lower surgical site infection rates (12), suggesting that minimizing the reopening area may be advantageous. Potential risks of this approach include an increased likelihood of disorientation, thereby making the procedure more dependent on the accuracy of navigation systems. In addition, managing massive intraoperative bleeding may be challenging. In such situations, conversion to a conventional open approach may be required.

It has been reported that the majority of recurrent GBMs present as local recurrences, accounting for approximately 75%–79% of cases (13, 14). Although the survival benefit of repeat resection for recurrent GBM has not been firmly established, a high extent of resection for residual tumors < 1 cm^3^ has been reported to improve survival (15), and a recent meta-analysis indicated that surgical intervention may be beneficial in selected cases (9). Thus, there appears to be a substantial number of cases that could be considered candidates for this approach.

In recent years, novel treatments for recurrent GBM, such as oncolytic virus therapy (e.g., Delytact®), have also been introduced (16, 17). These therapies often require stereotactic or localized administration, and the development of local treatment strategies other than resection is expected to continue. The present approach may also facilitate localized therapies. Furthermore, this technique may have potential utility not only for GBM but also for other intraparenchymal tumors and atypical meningiomas.

This report describes a single case and demonstrates the feasibility of the proposed approach. However, its safety and effectiveness require further validation. Additional cases and systematic evaluations are necessary to establish the clinical role of this technique.

## Conclusions

In this report, we describe the successful resection of a locally recurrent intracranial lesion using a minimally invasive multi-port surgical approach that utilized a pre-existing resection cavity from the initial surgery. This case demonstrates that a port-based technique leveraging an existing resection cavity may be a feasible surgical option for superficially located recurrent intracranial lesions. With further accumulation of cases, this approach has the potential to become a favorable alternative to conventional surgery by improving operative efficiency and reducing wound-related complications, thereby facilitating less invasive and more effective surgical management.

## Data Availability

The original contributions presented in the study are included in the article/Supplementary Material, further inquiries can be directed to the corresponding author.
